# Effect of Foliar Application of Process Water from Hydrothermal Carbonization of Sugar-Energy Byproducts on Tomato Plant Growth and Chlorophyll Content

**DOI:** 10.1021/acsomega.6c06470

**Published:** 2026-10-29

**Authors:** Suelen A. Mondek, Gabriela A. Nogueira, Isabela C. Constantino, Odair P. Ferreira, Márcia C. Bisinoti, Altair B. Moreira

**Affiliations:** † Sao Paulo State University (UNESP), Institute of Biosciences, Humanities and Exact Sciences (IBILCE), Department of Chemistry and Environmental Sciences (DQCA), Laboratory of Environmental Sciences Studies (LECA), 2265 Cristovao Colombo St., Sao Jose do Rio preto, São Paulo 15054-000, Brazil; ‡ State University of Londrina (UEL), Department of Chemistry, Advanced Functional Materials Laboratory (LaMFA), Celso Garcia Cid Highway, PR 445, Km 380, Londrina 86055-900, Brazil

## Abstract

Process water (PW) from hydrothermal carbonization (HTC) of agro-industrial residues is rich in carbon and nutrients yet underexplored as a foliar fertilizer. Two PWs from HTC of sugar cane bagasse and vinasse using H_3_PO_4_ (PW_A_) and KOH (PW_B_) were employed as foliar treatments (at 50, 100, and 300 mg TOC L^–1^) with/without commercial fertilizer (CF) on tomato growth. PW_A_ exhibited greater hydrophobicity, whereas PW_B_ was more hydrophilic and richer in organic compounds. Plant growth and chlorophyll responses were strongly dose-dependent, with phytotoxicity observed at higher TOC levels (300 mg TOC L^–1^), whereas 100 mg TOC L^–1^ yielded optimal biostimulatory effects. PW_B_ at 100 mg TOC L^–1^ with CF produced the largest growth response, increasing shoot length by up to 29% relative to the commercial fertilizer control. PW_A_ at the same concentration with CF increased shoot length by 11% compared to the commercial fertilizer control. Relative to distilled water and fertilizer controls, chlorophyll content increased by 60% and 49% with PW_A_ (at 100 mg TOC L^–1^), and by 11% and 9% with PW_B_, respectively. These results indicate that foliar application of PW at 100 mg TOC L^–1^ is promising as a foliar fertilizer supplement to stimulate chlorophyll production and tomato plant growth.

## Graphical abstract

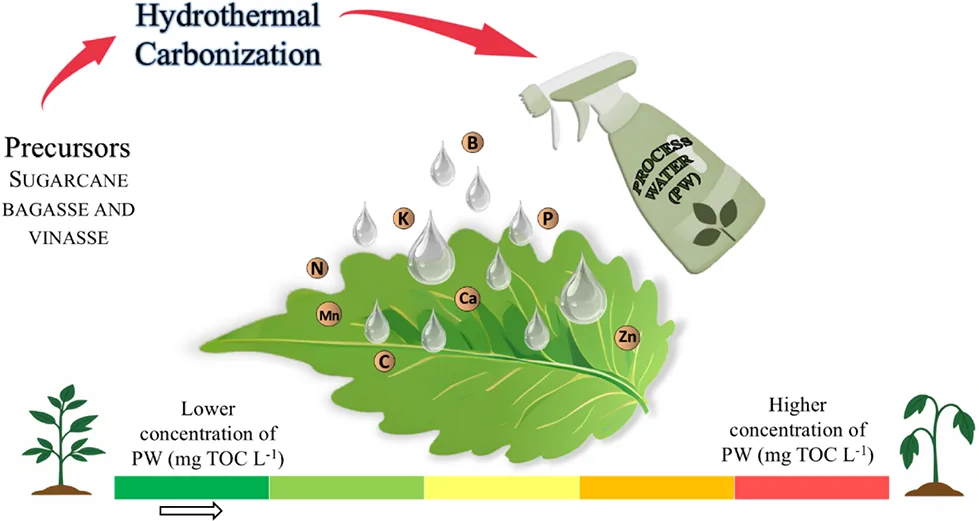

## 1 Introduction

The global population growth has driven the intensive use of fertilizers to meet crop nutritional demands and enhance agricultural productivity.^1^ However, the improper application of fertilizers can result in environmental effects such as eutrophication of aquatic ecosystems, and chemical and biological properties of soils alterations.^2–4^ With approximately 33% of global soils degraded due to unsustainable practices, developing eco-friendly agricultural inputs has become an urgent priority.^5,6^ Repurposing waste streams as agricultural inputs directly aligns with circular economy principles and contributes to the United Nations Sustainable Development Goals (SDGs), particularly the eradication of hunger (SDG 2) and the promotion of sustainable production and consumption patterns (SDG 12).^7,8^

Hydrothermal carbonization (HTC) is a thermochemical conversion process carried out under moderate temperature (180–250 °C) and self-generated pressure,^9^ and has emerged as an efficient strategy for the valorization of agro-industrial residues, such as vinasse, sugar cane bagasse, sewage sludge, agricultural straw, microalgae, and food waste.^10–15,82^ Vinasse and sugar cane bagasse are large-scale byproducts generated during sugar cane processing. It is estimated that for each metric ton of sugar cane processed, approximately 280 kg of bagasse are produced, while 10 to 14 L of vinasse are generated per liter of ethanol produced.^16^ HTC provides an alternative for managing these residues, yielding three fractions: hydrochar (HC), process water (PW), and a gaseous phase. Depending on the reaction medium, acidic (e.g., H_3_PO_4_, PW_A_) or basic (e.g., KOH, PW_B_), distinct physicochemical characteristics are obtained for both HC and PW.^10,11,17^

HC has been extensively studied for its potential as a soil conditioner and complexing agent for the remediation of contaminated compartments, highlighting its applicability in the agricultural sector.^10,11,18,19^ Conversely, the use of PW as a foliar-applied fertilizer remains underexplored, despite the liquid process phase retaining a high concentration of dissolved mineral nutrients and organic compounds, such as potassium, calcium, magnesium, iron, and zinc.^17^ This composition supports its potential as an alternative foliar fertilizer source.

Foliar fertilization is a complementary approach to soil fertilization, offering faster nutrient absorption and reducing losses through leaching and volatilization, while enabling the direct supply of macro- and micronutrients to plants, thereby enhancing nutrient assimilation and use efficiency.^20,21^ The application of PW from HTC as a liquid fertilizer in agricultural systems, with direct soil application, has been more extensively investigated using a wide variety of biomasses, including poultry litter, poplar sawdust, wheat straw, cattle manure, and food waste, among others, demonstrating improvements in plant growth, biomass accumulation, and nutrient uptake by roots.^22–26^

For example, PW from the HTC of forestry residues (poplar sawdust) and agricultural residues (wheat straw) applied to paddy rice cultivation increased grain yield by 4.3–26.0%.^23^ Moreover, its composition has been explored as a source of humic substances with metal-chelating properties,^27^ reinforcing its potential as a fertilizer. Other studies have investigated PW for applications in anaerobic digestion, resulting in increased methane production,^28^ and in phosphorus recovery from coffee grounds,^29^ highlighting its broad applicability in agricultural and environmental systems.

Recently, PW from the HTC of sugar cane bagasse and vinasse in acidic media (H_2_SO_4_) was applied by our research group as an additive to foliar fertilizers in tomato and maize cultivation, as described in patent BR 10 2025 003036-5.^30^ Although HTC processes for biomass valorization and agricultural applications are well established, the physiological impacts of foliar-applied PW derived from distinct reaction media remain to be fully elucidated, creating a growing demand for novel applications within the circular economy framework, as well as for the proper management of PW.

Within this context, the present study seeks to contribute for sustainable development by valorizing PW from the HTC of sugar cane bagasse and vinasse. By repurposing PW from HTC as a foliar biostimulant, this approach offers a dual solution for the sugar-energy sector: mitigating environmental burdens associated with wastewater management while providing sustainable input for agricultural systems. Accordingly, the study evaluated the feasibility of applying PW as a foliar treatment in tomato plants. Two types of PW, obtained by HTC from sugar cane byproducts (bagasse and vinasse) under acidic and basic reaction conditions, were used. Foliar applications were performed both independently and in conjunction with a commercial fertilizer (CF), with effects assessed through measurements of plant growth parameters and chlorophyll content.

## 2 Materials and Methods

### 2.1 PW Production

Two PWs were obtained by HTC of a sugar cane bagasse and vinasse mixture at a solid-to-liquid ratio of 1:20 (w/v) in stainless-steel reactors, as described previously.^31^ Briefly, bagasse was combined with vinasse in the presence of specific additives: PW_A_ was produced by adding 4.0% (v/v) phosphoric acid (*H*_3_ *PO*_4_, 85%, Sigma-Aldrich), while PW_B_ was produced by adding 10 mL of a 4.0 mol L^–1^ potassium hydroxide solution (KOH, 85%, Sigma-Aldrich) to achieve a final concentration of 0.1 mol L^–1^ in the reaction mixture. Hydrothermal reactions were carried out in a muffle furnace under static conditions at 230 ± 10 °C for 13 h. After completion of the HTC, the reactors were removed from the furnace and immediately cooled in an ice bath to room temperature. The solid phases (HCs) were separated from the liquid phases (PWs) by vacuum filtration using qualitative filter paper and cellulose acetate membranes (0.45 μm, Millipore). The sugar cane bagasse used in this study was previously registered in the *Sistema Nacional de Gestão do Patrimônio Genético e Conhecimentos Tradicionais Associados* (SisGen) under registration number A0018C2.

### 2.2 PW Characterizations

PW_A_ and PW_B_ were lyophilized (Thermo Electron Corporation, MODULYO D-23, United Kingdom) to enable solid-state characterization using different techniques. For the PW_B_, an additional dialysis step was required before characterization due to its highly hygroscopic nature. Specifically, PW_B_ was purified using dialysis membranes (Spectra/Por 6, MWCO = 1 kDa) and immersed in distilled water until constant conductivity was achieved (14.28 μS cm^–1^). The total organic carbon (TOC) concentration was determined using a Total Organic Carbon Analyzer (Shimadzu, TOC-VCSN, Japan), whereas pH was measured with a pH meter (Marconi, PA 200, Brazil). Molecular fluorescence spectra were acquired from solutions containing 5.0 mg TOC L^–1^ according to the analytical curve (Figure S1) in NaHCO_3_ (0.05 mol L^–1^, pH 8.0) using a spectrofluorometer (Cary Eclipse, Varian, Australia) in excitation–emission matrix (EEM) mode, with both excitation and emission wavelengths ranging from 200 to 600 nm, a slit width of 5 nm, and a scan speed of 1200 nm min^–1^, with the detector voltage set to 700 V.

In the lyophilized PWs, the carbon (C), nitrogen (N), and hydrogen (H) contents were determined using a CHN elemental analyzer (Fisons, EA 1108, United States of America). Ash content was determined by thermogravimetric analysis (TGA; PerkinElmer, TGA-4000, United States of America) using approximately 10 mg of each PW, under a synthetic air atmosphere, with an initial stabilization at 30 °C for 1 min, followed by heating to 900 °C at a rate of 10 °C min^–1^. Functional groups were identified by Fourier-transform infrared spectroscopy (FTIR; PerkinElmer, Spectrum Two, United States of America), with PWs analyzed using an attenuated total reflectance (ATR) accessory, and spectra collected in the 4000–400 cm^–1^ range, at a resolution of 4 cm^–1^, averaging 30 scans.

### 2.3 Foliar Application of PW on Tomato Plants

The foliar application procedure followed the methodology previously described.^31^ Tomato plants (*Solanum lycopersicum* *L*.) were grown individually in pots (13 cm height × 7 cm diameter) containing 700 g of Ultisol collected from the rural area of Quatá, São Paulo State, Brazil (20°48′19.79″S, 49°19′43.51″W). The soil was sieved through a 2.72 mm mesh, pH-adjusted to 6.0 with calcium oxide (CaO), and its physicochemical properties are shown in Table S1. Each treatment had five replicates, and plants were maintained in a climate-controlled chamber (TE-4002, Tecnal, Brazil) at 26 ± 2 °C, 55% relative humidity, and a 16/8 h light/dark photoperiod for 35 days.

Twelve days after sowing, NPK 20:20:20 fertilizer was applied directly to the soil in all pots, and foliar spraying was initiated. Foliar treatments consisted of three doses of PWs based on TOC content (50, 100, and 300 mg L^–1^), applied at seven-day intervals. Two control groups were established: (i) WC (negative control), sprayed with distilled water only, and (ii) FC (positive control), treated with commercial fertilizer (CF) diluted at 500:0.75 (v:v) (Titanium Completo–Solo Rico, composition: 5.0% N, 8.0% P, 5.0% K, 1.0% Ca, 0.6% Mg, 0.04% B, 0.02% Cu, 0.5% Mn, 1.0% Zn, supplemented with 28.0% amino acids).

The six PW foliar treatments, differentiated by TOC concentration and combination with CF, were designated as follows: PW at 50 mg TOC L^–1^ (T-50 and T-50F), PW at 100 mg TOC L^–1^ (T-100 and T-100F), and PW at 300 mg TOC L^–1^ (T-300 and T-300F). Treatments with the suffix “F” involved PW combined with the CF, while those without the suffix received PW exclusively. For exclusive PW applications, PW_A_ (initial pH 3.05 ± 0.06) was neutralized with 10 drops of 0.1 mol L^–1^ KOH to pH 6.85 ± 0.05. This adjustment was not required for PW_B_ because its initial pH was already near-neutral (6.77 ± 0.04). Thus, both PW solutions were applied at equivalent, nonphytotoxic pH levels, ensuring that observed physiological differences were attributable to the specific chemical composition of the HTC fractions rather than pH artifacts. Furthermore, KOH was selected as the neutralizing agent to avoid introducing nonessential cations (such as Na^+^) into the foliar treatment.

#### 2.3.1 Parameters for Analysis in Tomato Plant Growth Studies

At the end of the experiment (35 days), root length and shoot length (cm) were measured using ImageJ software. Subsequently, shoots and roots were separated and subjected to drying in an oven at 105 °C for 24 h (Tecnik’s, NL80/10CEL), followed by storage in a desiccator and weighing to determine dry weight in an analytical balance (Shimadzu, ATX224R, Japan). The chlorophyll index (Falker chlorophyll index–FCI) was measured using a chlorophyll meter (clorofiLOG, Falker, Brazil), calculated as the average of three readings taken at distinct points on the middle third of the same nonsenescent leaf.

### 2.4 Data Analysis

The effects of PWs on tomato plant growth parameters and chlorophyll content were analyzed using one-way ANOVA, with F-tests conducted at a significance level of 0.05. Tukey’s post hoc test was employed for multiple mean comparisons (α = 0.05). All analyses were performed using OriginPro 2025 software (OriginLab, Northampton, Massachusetts, USA). The figures were designed by the authors using Canva Pro (Sydney, Australia). All plant growth parameters were evaluated at the end of the 35-day experimental period; therefore, the statistical comparisons were performed among treatments within the respective parameter at this final time point. Different letters assigned to the means indicate statistically significant differences among treatments according to Tukey’s test (α = 0.05).

## 3 Results and Discussion

### 3.1 Characterizations of PWs

PW_A_ exhibited a pH of 3.05 ± 0.06, whereas PW_B_ showed a pH of 6.77 ± 0.22. Besides that, TOC concentrations for PW_A_ and PW_B_ were 38.4 and 47.1 g L^–1^, respectively, which are associated with biomass conversion processes during HTC, such as hydrolysis, dehydration, condensation, and aromatization.^32–34^ Previous studies have reported that these thermochemical reaction pathways enable the formation of various soluble organic compounds, including organic acids (such as lactic, formic, and levulinic acids), benzenetriol, and other low-molecular-weight metabolites.^32,35–38^ PW produced under the same reaction parameters and precursors as PW_A_ exhibited relatively lower pH (2.47 ± 0.19) and TOC (25.2 g L^–1^) values.^17^ This variation can be attributed to the inherent heterogeneity of sugar cane bagasse and vinasse, which has also been observed in other studies employing sugar cane bagasse and/or vinasse.^39,40^

Elemental composition of the PWs was altered depending on the additive used in the reaction media (Table 1). An 8% increase in carbon content, accompanied by reductions of 16 and 28% in hydrogen and nitrogen contents, respectively, was observed for PW_A_ compared to PW_B_ (Table 1). These variations resulted in lower atomic H/C ratios for the PW_A_ relative to PW_B_, indicating higher aromaticity, degree of carbonization, and chemical stability, which contribute to the increased hydrophobicity observed in PW_A_.^36,41,42^ Furthermore, lower atomic H/C ratios are frequently correlated with higher fixed carbon content.^19,43^ Atomic H/C ratios similar to those obtained in this study (0.9 and 1.1 for PW_A_ and PW_B_, respectively) have been reported in the literature (1.1–1.5) for promising liquid fertilizers.^44,45^ Moreover, the increase in the atomic C/N ratio suggests a decrease in nitrogen-containing groups, which is also associated with materials exhibiting a higher carbonization degree and hydrophobicity.^43,46^

**Table 1:** CHN Elemental Analysis, Atomic Ratios, and PW_A_ and PW_B_ Ash Content

	Elemental composition				Atomic Ratios	
Samples	C (%)	H (%)	N (%)	Ashes (%)	H/C	C/N
PW_A_	58.3	4.4	2.1	50.0	0.9	32.8
PW_B_	54.1	5.2	2.8	11.1	1.1	22.2

TG and DTG curves (first derivative of the TG curve) profiles of PW_A_ revealed the first thermal weight loss event occurring up to 150 °C (7.3%; Figure 1a), attributed to the loss of adsorbed water and volatile compounds such as acetic and formic acids formed during HTC.^33,34^ The second thermal event, between 150 and 250 °C (16.8%), was associated with the degradation of low molecular weight organic compounds. Between 250 and 350 °C (10.1%), the weight loss corresponded to the decomposition of aromatic structures and more complex organic compounds, such as humic and fulvic acids, as reported in the literature.^47^ On the other hand, the final thermal mass loss event (12.4%) occurring between 350 and 600 °C was related to the carbonization of nonvolatile organic residues, a fraction rich in fixed carbon. Above 600 °C, no significant weight variation was observed. The residue was thus considered the ash content of the sample, resulting from the complete oxidation of the carbonaceous phase and stabilization of the inorganic fraction, as also observed in previous studies.^48,49^ PW_A_ exhibited organic and inorganic contents of 39.2% and 50.0%, respectively (Table S2).

**Figure 1:**
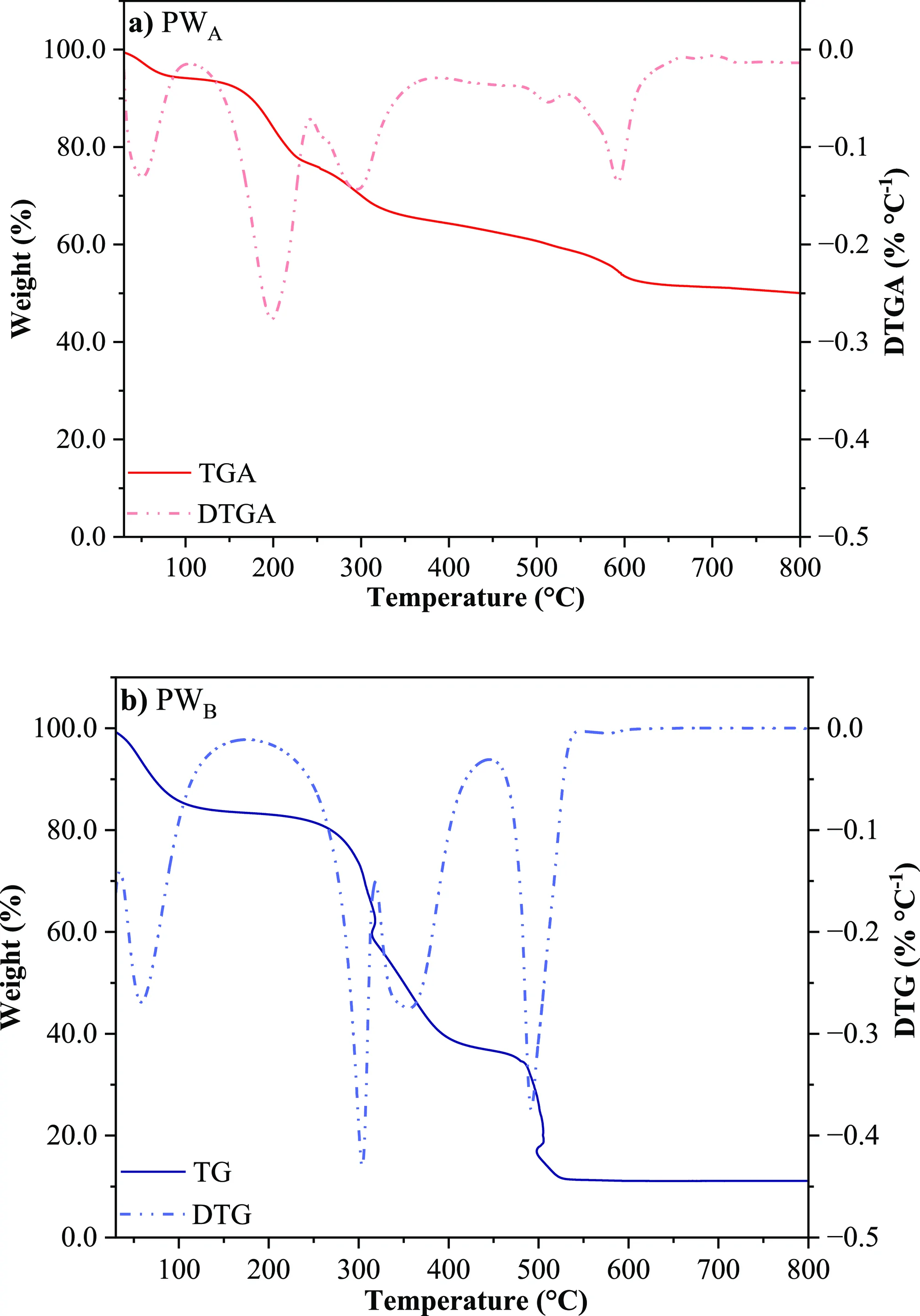
Thermogravimetric curves (TG) and first derivative (DTG) for a) PW_A_ and b) PW_B_.

PW_B_ likewise displayed four thermal weight loss events (Figure 1b). However, it presented an organic content 1.8 times higher (72.6%; Table S2) and an inorganic content 4.5 times lower (11.1%; Table 2) compared to PW_A_. The lower ash content of PW_B_ can be attributed to the dialysis process applied to this sample due to its initially high hygroscopicity. Dialysis removes soluble ions and small molecules, including inorganic salts that would otherwise contribute to ash content if retained.^50^ Additionally, an increasing trend in the onset decomposition temperature for each thermal event was observed, with increases of 65, 60, and 46 °C for the second, third, and fourth thermal events, respectively, indicating enhanced thermal stability of PW_B_ relative to PW_A_. Furthermore, PW_B_ exhibited adsorbed water content (16.3%; Table S2) that was 2.2 times greater than that observed for PW_A_, indicating higher surface hydrophilicity.

**Table 2:** Growth Performance of Tomato Plants after 35 Days of Foliar Application with PW_A_ and PW_B_ at Concentrations of 50, 100, and 300 mg TOC L^–1^, Applied with (+F) and without (−F) Supplemental Fertilizer, Compared to Controls (WC: Water Control; FC: Fertilizer Control)^a^

			Lengths (cm)		Dry weight (g)	
Samples		Number of leaves	Root	Shoot	Root	Shoot
**WC**	PW_A_	19.8 ± 0.8	4.6 ± 0.5	19.2 ± 0.8	0.18 ± 0.01	0.44 ± 0.05
	PW_B_	16.4 ± 2.1	4.2 ± 0.8	17.0 ± 1.0	0.16 ± 0.01	0.44 ± 0.03
**FC**	PW_A_	21.2 ± 1.3	5.0 ± 0.7	20.5 ± 1.0	0.19 ± 0.01	0.49 ± 0.02
	PW_B_	19.6 ± 1.5	4.0 ± 0.7	19.2 ± 0.8	0.17 ± 0.01	0.49 ± 0.04
**T-50**	PW_A_	31.0 ± 2.8^ab^	5.1 ± 0.2	21.2 ± 1.4^b^	0.19 ± 0.01	0.50 ± 0.02
	PW_B_	28.2 ± 2.2^ab^	4.2 ± 0.8	21.0 ± 2.5	0.18 ± 0.01	0.51 ± 0.06
**T-50F**	PW_A_	30.4 ± 1.8^ab^	5.0 ± 0.7	21.3 ± 0.9^b^	0.19 ± 0.04	0.51 ± 0.03^b^
	PW_B_	30.2 ± 3.6^ab^	5.4 ± 0.9	21.6 ± 1.1^b^	0.19 ± 0.01	0.51 ± 0.05
**T-100**	PW_A_	24.6 ± 1.7^b^	4.7 ± 0.67	20.8 ± 0.8	0.18 ± 0.02	0.50 ± 0.01
	PW_B_	25.4 ± 4.5^ab^	4.0 ± 0.7	20.5 ± 3.0	0.16 ± 0.03	0.49 ± 0.01
**T-100F**	PW_A_	27.6 ± 3.4^ab^	4.7 ± 0.4	21.4 ± 0.6^b^	0.20 ± 0.01	0.54 ± 0.03^b^
	PW_B_	26.2 ± 3.2^ab^	4.5 ± 0.9	21.9 ± 2.0^b^	0.20 ± 0.02^b^	0.55 ± 0.05^b^
**T-300**	PW_A_	23.0 ± 3.2	4.1 ± 0.5	19.8 ± 0.6	0.19 ± 0.01	0.46 ± 0.02
	PW_B_	17.4 ± 1.5	3.5 ± 1.0	15.4 ± 2.2	0.12 ± 0.01^ab^	0.36 ± 0.02^a^
**T-300F**	PW_A_	19.2 ± 1.5	4.0 ± 0.8	19.9 ± 0.7	0.20 ± 0.01	0.47 ± 0.03
	PW_B_	17.4 ± 1.1	2.8 ± 0.8	15.4 ± 2.1	0.12 ± 0.01^ab^	0.39 ± 0.05^a^

a Evaluatedparameters included leaf number, leaf length, and dry weight of rootsand shoots. Data are presented as mean ± standard deviation (SD). Statistical significance was assessed at α = 0.05. Different letters indicate significant differences according to post-hoc comparisons:‘a’ denotes significant differences relative to the FC, and ‘b’denotes significant differences relative to the WC.

FTIR spectrum of PW_A_, recorded in the range of 4000–400 cm^–1^ (Figure 2), exhibited a broadband between 3700 and 3000 cm^–1^, with maximum intensity at 3290 cm^–1^, assigned to O–H stretching vibrations of phenolic and alcoholic groups. Low-intensity bands at 2986, 2938, and 2886 cm^–1^ were associated with the symmetric and asymmetric stretching vibrations of CH_3_, CH_2_, and CH groups. In addition, the band at 1713 cm^–1^ was attributed to the C=O stretching of carboxylic acids, aldehydes, and ketones, whereas the bands at 1561 and 1505 cm^–1^ corresponded to C=C stretching of aromatic compounds.^51,52^ Furthermore, the bands at 1453 and 1396 cm^–1^ were related to C–H bending, and the band at 1199 cm^–1^ to C–OH stretching in phenolic groups.^10,19,53,54^ In the region between 1140 and 770 cm^–1^, overlapping bands were observed, corresponding to C–O–C vibrations and deformations, as well as Si–O–Si and P–O vibrations. Previous studies have indicated the presence of silicon in sugar cane bagasse, attributed both to residual sand particles within the fibers and to silicon naturally occurring in the cell wall structure.^11,55^ Moreover, the presence of H_3_PO_4_ in the reaction medium enables the formation of P–O bonds.

**Figure 2:**
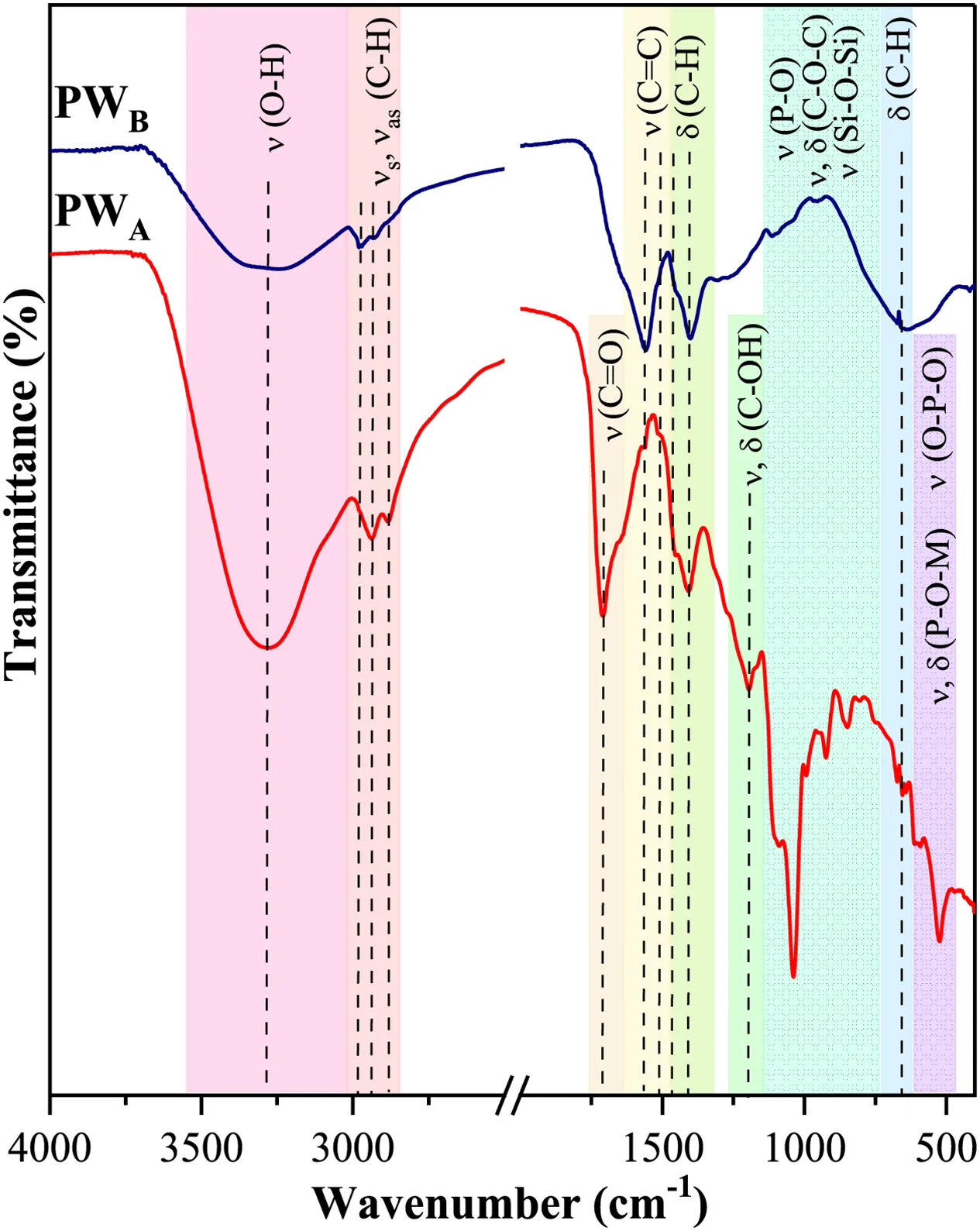
Infrared spectra for PW_A_ and PW_B_.

PW_A_ composition, obtained from the HTC of sugar cane bagasse, vinasse, and H_3_PO_4_, suggests that the inorganic constituents present in vinasse, such as calcium, iron, magnesium, potassium, phosphorus, among others, contributed to the formation of phosphorus–oxygen–metal (P–O–M) bonds.^40^ However, due to the high concentration of inorganic compounds in vinasse and aggregation in PW, it is not feasible to distinguish which specific metal is responsible for the observed band, as bonds with more than one metal are likely to occur, resulting in band overlapping.^56^ In addition, the bands between 680 and 660 cm^–1^ were attributed to C–H angular deformations, whereas the range between 600 and 470 cm^–1^ was also associated with overlapping stretching modes and the vibrations of O–P–O and P–O–M bonds.^10,57^ Phosphorus incorporation into PW_A_ may result in a foliar fertilizer with the potential to enhance energy transfer and cellular metabolism in plants, as well as to promote increased photosynthetic activity and the rapid correction of nutrient deficiencies.^58^ The spectral features observed in the solid obtained by lyophilizing the PW are consistent with functional groups reported in the literature for substances present in HTC byproducts, reinforcing the chemical complexity of the material produced.^59,60^

FTIR spectrum of PW_B_ (Figure 2) revealed spectral characteristics distinct from those of PW_A_. Notably, the bands associated with C=O (1713 cm^–1^) and C–OH (1199 cm^–1^) bonds exhibited a pronounced decrease in intensity. These findings suggest that the addition of KOH during the HTC process promoted the deprotonation of phenolic and carboxylic acid groups and their derivatives.^61–64^ Furthermore, bands attributed to O–P–O and P–O–M bonds (600–470 cm^–1^) were not identified, as the additive employed in the HTC process was KOH, supporting the hypothesis that such bonds in PW_A_ were formed due to the presence of H_3_PO_4_. It has been reported that KOH can generate humic acid–like products during HTC, resulting in spectral profiles similar to those obtained in the present work, with bands corresponding to O–H, C–H, C=C, among others.^65^

Fluorescence spectra of PWs (Figure 3) exhibited distinct features, reflecting the influence of the different additives used in the HTC process. The sample processed under acidic HTC conditions (PW_A_) displayed higher fluorescence intensity, with excitation peaks around 230 nm and emission in the range of 400–410 nm. These results suggest the presence of easily excitable fluorophores,^66^ characterized by lower structural complexity.

**Figure 3:**
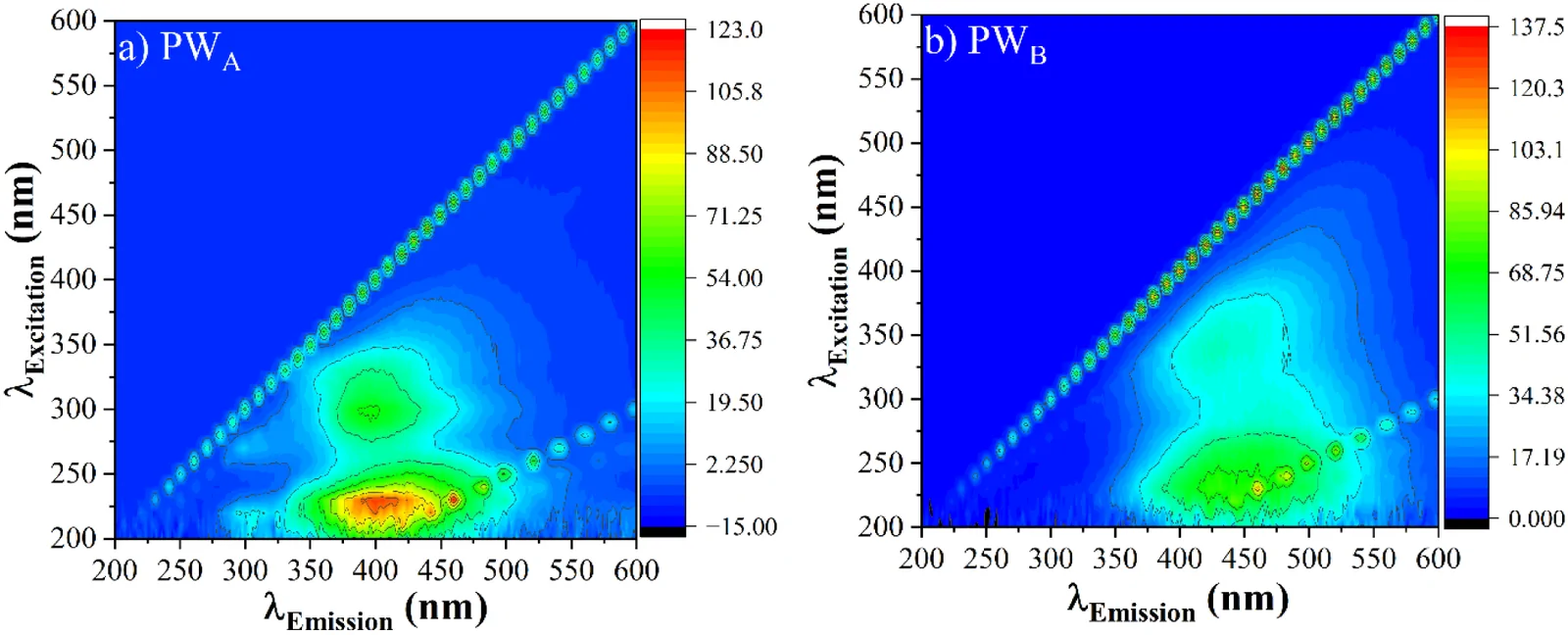
Fluorescence spectra in the EEM mode of the PW solutions at 5.0 mg TOC L^–1^: a) PW_A_ and b) PW_B_.

On the other hand, PW_B_, processed under basic HTC conditions, exhibited excitation peaks in the same range (∼230 nm) but with slighted, red-shifted emission at longer wavelengths (420–440 nm) and lower fluorescence intensity. This behavior can be attributed to the greater structural complexity and heterogeneity of the organic compounds present.

In general, PW_A_ and PW_B_, obtained by the HTC process using sugar cane bagasse and vinasse, exhibited distinct physicochemical characteristics, which were strongly influenced by the H_3_PO_4_ and KOH additives, respectively. PW_A_ displayed higher hydrophobicity and a greater fixed carbon content, which can be attributed to increased aromaticity and a reduction in oxygen-containing functional groups. In addition, the presence of functional groups associated with the interaction between vinasse-derived metals and phosphorus from the acidic additive was observed. Conversely, PW_B_ exhibited higher hydrophilicity and a greater content of organic compounds, likely due to the dialysis process, which removed low-molecular-weight ions. The use of KOH promoted the deprotonation of phenolic and carboxylic acid groups, as well as greater thermal stability when compared to PW_A_. The EEM fluorescence spectra corroborated the observed structural differences, with the PW_A_ exhibiting higher fluorescence intensity than PW_B_.

### 3.2 Effects of PW Foliar Application on Tomato Plant Growth

Foliar application of PWs derived from HTC of sugar cane bagasse and vinasse demonstrated that both samples, PW_A_ and PW_B_, possess the potential to stimulate tomato plant growth, particularly at the lower concentrations (50 and 100 mg TOC L^–1^). These effects were observed when applied individually or in combination with CF, showing significant improvements compared to both WC and FC (*p* ≤ 0.05) (Table 2).

The treatment with PW_B_ at a concentration of 100 mg TOC L^–1^ (T-100) produced the highest stimulation of plant growth, with increases of up to 29% in shoot length and 25% in root dry weight (Table 2). Similarly, PW_A_ at the same concentration also exhibited positive effects, resulting in increases of 11% in shoot length and 23% in root dry weight. The combined application of PW_B_ with a CF (T-100F) showed comparable results, suggesting that PW can act synergistically with foliar fertilizers.

At 50 mg TOC L^–1^, the T-50F treatment with PW_B_ stood out, promoting an 84% increase in leaf number and a 27% increase in shoot length. A single application of PW_B_ (T-50) was also effective, yielding a 72% increase in leaf number. In turn, PW_A_ at the same concentration (T-50 and T-50F) led to gains of up to 56.6% in leaf number, demonstrating comparable results to those of the CF.

However, application at a higher concentration (300 mg TOC L^–1^) did not improve the evaluated parameters, with results largely similar to the controls. In some cases, such as root dry weight, a significant reduction (*p* ≤ 0.05) was observed, indicating a potential phytotoxic effect or a limitation in the foliar absorption capacity of the compounds present at higher doses.^17,26^

The increase in tomato plant growth observed (Table 2 and Figure S2) may be associated with the distinct physicochemical properties of the PWs. PW_B_, obtained under basic conditions, exhibited a lower C/N ratio (22.24), linked to a higher content of nitrogen-containing compounds (Table 1) and a predominance of hydrophilic compounds. These characteristics enhance solubility and, consequently, the efficiency of nutrient solution absorption through foliar application.^21,67^ Moreover, PW_B_ showed a higher presence of oxygen-containing functional groups, associated with a greater content of organic compounds that increase solubility in water and organic solvents.^68^

On the other hand, PW_A_, produced under acidic conditions, exhibited a higher C/N ratio (32.83) and an inorganic content of 50% (Table S2), suggesting the presence of high concentrations of minerals and greater hydrophobicity, which may have limited its absorption compared to PW_B_. Nevertheless, its effects on plant growth were positive and superior to the controls, likely because its mineral composition may have favored foliar uptake.^67,68^

Application of PW derived from HTC of sugar cane bagasse and vinasse as a nutrient source via foliar spraying in plant growth experiments remains scarcely explored in the literature,^84,85^ despite the liquid process phase retaining a high concentration of dissolved mineral nutrients and organic compounds.^17^ Studies involving PW obtained from HTC of different biomasses and additives support the findings of the present research.^17,23,24,26,46^

Evidence reported in other works indicates that the reaction media used in the HTC process directly affect the concentrations of macro- and micronutrients present in the PW.^17,35,46,83^ A study employing sugar cane bagasse and vinasse with the addition of H_3_PO_4_ (4.0% v/v), a similar approach to that used herein with PW_A_, albeit with a different application focus, evaluated PW toxicity in a marine environment (*Artemia salina*) and in soil through seed germination assays (corn, lettuce, and tomato). The chemical characterization of this PW revealed respectively the presence, at 50 and 100 mg TOC L^–1^: potassium (29.4 and 58.8 mg L^–1^), calcium (2.2 and 4.4 mg L^–1^), magnesium (4.4 × 10^–3^ and 8.8 × 10^–3^ mg L^–1^), iron (0.20 and 0.41 mg L^–1^), zinc (5.2 × 10^–3^ and 0.01 mg L^–1^) and phosphorus (168.1 mg L^–1^).^17^

Furthermore, research on acid-assisted HTC (HCl 0.5 mol L^–1^) revealed that approximately 83% of the phosphorus present in swine manure was leached into the PW, highlighting that a substantial fraction of the precursor’s phosphorus content is transferred to the liquid phase.^69^ Therefore, the increase in dry weight and plant growth observed from PW_A_ application may be attributed to the high phosphorus content detected in the PW (Figure 2), as phosphorus plays a pivotal role in plant development, contributing to photosynthesis, root and shoot growth, crop quality and yield improvement, as well as accelerating plant maturation.^70,71^

In addition, previous studies have identified several semivolatile organic compounds in PW_A_, including phenols, ketones, alcohols, nitrogen-containing compounds, organic acids, and esters,^35^ which, depending on their concentration, may exert beneficial effects on plant metabolism.^26^ In another case, PW or HC from HTC of microalgae (*Spirulina*) with the addition of KOH (1.0 mol L^–1^) contained 1018 mg TOC L^–1^ of TOC, 520 mg L^–1^ of potassium, 20 mg L^–1^ of phosphate, and 109 mg L^–1^ of ammonium ion.^43^ The presence of compounds such as phosphate, nitrogen, and potassium in samples obtained with KOH (1.0 mol L^–1^) during HTC has been reported to enhance plant development.^72^

Therefore, the growth responses observed in tomato plants treated with PW_B_ at concentrations of 50 and 100 mg TOC L^–1^ may be largely attributed to the presence of potassium, which plays an important role in regulating multiple physiological processes, including growth, yield, quality, and protection against abiotic stress.^73,74^ Furthermore, the concentration of PW applied had a direct influence on the growth parameters obtained (Table 2), with the 300 mg TOC L^–1^ treatment leading to a reduction compared with the control.

Consistent with these findings, a seed germination study using PW_A_ demonstrated that diluted concentrations (50 and 100 mg TOC L^–1^) stimulated root and shoot growth in maize and tomato seedlings, whereas higher concentrations (500 mg TOC L^–1^) inhibited seed germination in maize, lettuce, and tomato, corroborating the results observed in the present study.^17^

Applications of PW from HTC of cattle manure in hydroponic maize (*Zea mays* L.) cultivation, at dilutions of 1:30, 1:60, and 1:90 (v/v), showed that higher dilutions (1:60 and 1:90) reduced phytotoxicity, resulting in improved plant development and greater biomass accumulation. On the other hand, higher concentrations (1:30) induced toxicity, impairing growth.^26^ Although these results were obtained through nutrient solution application rather than foliar spraying, they reinforce the potential of PW derived from various biomass sources, when applied at appropriate concentrations, as effective nutrient sources, originating from both inorganic content and organic compounds, for plant growth, either as stand-alone inputs or as additives to foliar fertilizers.

PW concentrations above 300 mg TOC L^–1^ are often associated with elevated levels of phytotoxic compounds, such as organic acids, phenols, and aldehydes, generated during HTC.^75,76^ These compounds can inhibit plant growth due to their toxicity, whereas moderate concentrations of phenolic compounds, including flavonoids and phenylpropanoids, have been linked to enhanced plant development and protection against both biotic and abiotic stresses.^77^ These findings highlight the importance of controlling both the concentration and chemical composition of PW to optimize plant growth, suggesting that adjustments to the HTC process and application strategy.

Another relevant aspect concerns a hypothesized mechanism (Figure 4) through which PW may influence tomato plant development. Based on the physicochemical characteristics of the PWs and previous literature, compounds present in these solutions may potentially interact with the leaf surface and be absorbed through the cuticle and/or stomata.^21,67^ However, foliar nutrient uptake was not directly measured in the present study, and therefore this proposed pathway should be regarded as a hypothesis for future.

**Figure 4:**
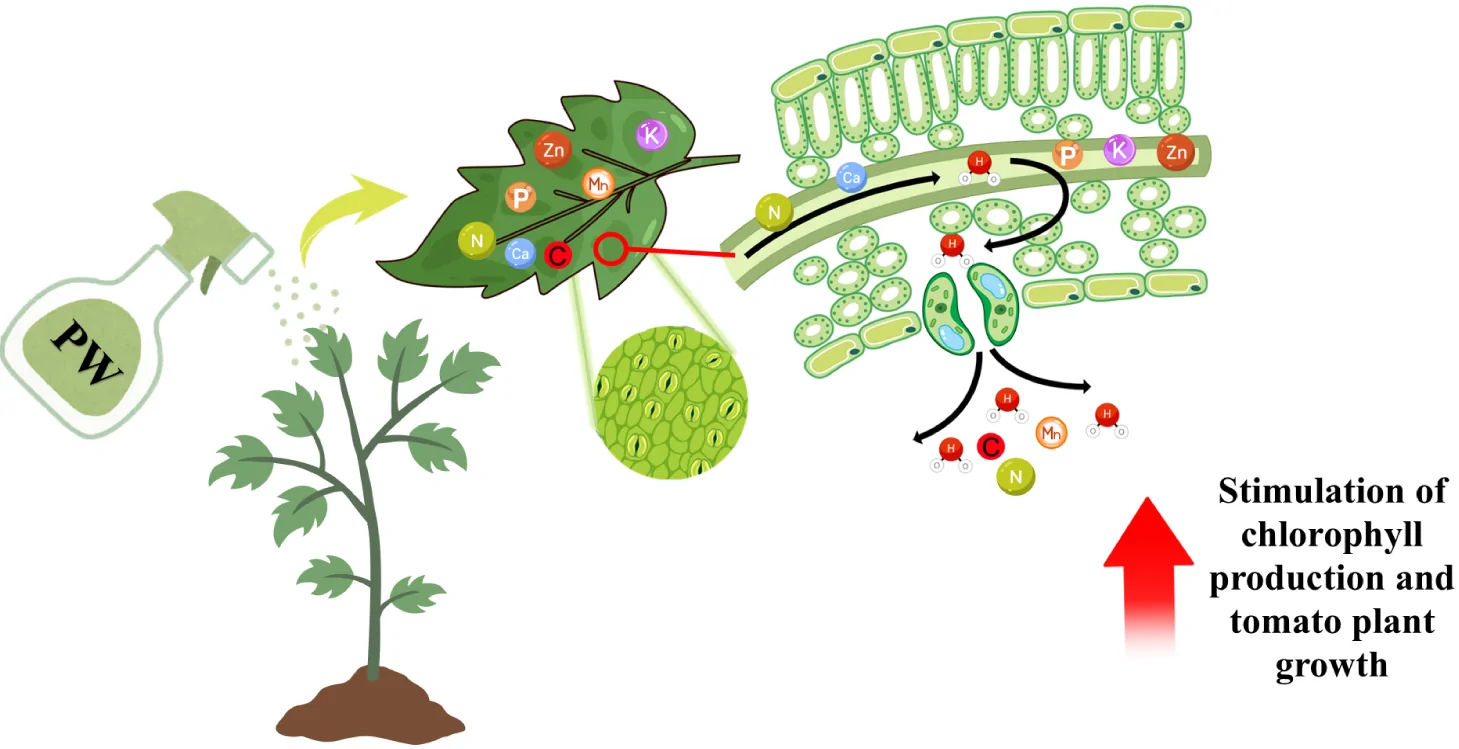
Hypothesized representation of the potential interaction and foliar uptake of compounds present in PW by tomato leaves.

Based on the characteristics observed in this study (Section 3.1), hydrophilic solutions with higher dissolved organic content may diffuse more readily through cuticular pores and stomata, whereas those with higher fixed carbon content and greater hydrophobicity may follow different absorption and retention dynamics on the leaf surface. Although foliar nutrient uptake was not measured, the observed effects suggest that the chemical composition of each PW influenced its interaction with the leaf surface and its potential as a fertilizer.

### 3.3 Chlorophyll Index in Tomato Leaves

Foliar applications of PW_A_ and PW_B_ at concentrations of 50 and 100 mg TOC L^–1^ resulted in significant increases in the foliar chlorophyll index (FCI) compared with the control treatments WC and FC (*p* ≤ 0.05) (Table S3). The highest chlorophyll accumulation values were obtained with PW_A_ at 100 mg TOC L^–1^ combined with CF (T-100F), with increases of 60 and 49% relative to WC and FC, respectively. Under the same conditions, PW_B_ achieved increases of 11 and 9% relative to WC and FC, respectively. Conversely, higher concentrations (300 mg TOC L^–1^) of PW_B_ did not yield further improvements in FCI, showing values statistically comparable to the controls (Figure 5).

**Figure 5:**
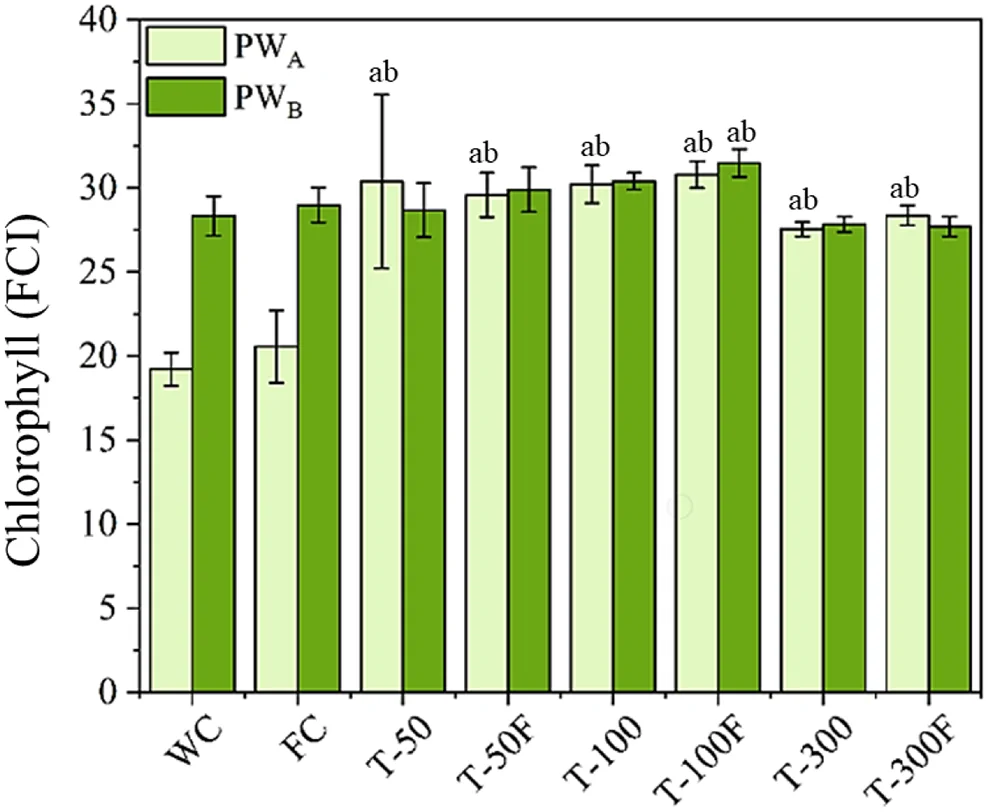
Total chlorophyll indexes (FCI) for tomato leaves at the end of the growth experiment with PW_A_ and PW_B_ application. Columns represent mean values ± SD. Statistical differences (α = 0.05) indicated by letters “a” indicates significant differences from the commercial fertilizer control (FC), and “b” indicates differences from the distilled water control (WC).

Similarly to the present study, the application of PW from HTC of cattle manure biomass, assessed as a potential fertilizer solution for hydroponic maize (*Zea mays* L.) growth, resulted in chlorophyll contents equivalent to the positive control obtained with a conventional nutrient solution (SPAD chlorophyll index of 33.2).^26^ The SPAD index observed is consistent with the FCI obtained in this study, demonstrating that the application of PW_A_ and PW_B_ also yielded values close to 30.0 in tomato leaves when applied at appropriate concentrations, both in the presence and absence of nutrient supplementation, compared with the control groups, thus contributing to the plant’s energy metabolism.

The FCI at 300 mg TOC L^–1^ remained comparable to that observed for WC. Previous studies suggest that potential phytotoxic effects may be associated with the predominance of nitrogen in the ammonium form, whose assimilation is less efficient for photosynthetic activity than inorganic nitrogen.^17,22,84^ Supporting this hypothesis, it was observed that PW obtained under reaction parameters identical to those of PW_A_ contained 9.2% nitrogenous compounds, including 2,6-dimethylpyrazine, a nitrogen-containing heterocyclic molecule.^35^ Even after dilution of PW in distilled water, partial protonation due to water autoionization may have occurred, leading to the formation of ammonium ions.^78^

Moreover, a study on the phytotoxicity and nutritional behavior of PW derived from the HTC of poultry litter confirmed that NH_4_^+^ is the main form of nitrogen available in such solutions,^22^ a result consistent with findings from PW obtained from HTC of microalgae, such as *Spirulina* and *Chlorella*, at 300 °C in the presence of KOH (1.0 mol L^–1^).^46^

Therefore, the results indicate that PWs derived from HTC of sugar cane bagasse and vinasse, produced in acidic (H_3_PO_4_) or basic (KOH) media at a concentration of 300 mg TOC L^–1^, may not favor tomato plant development and, in some cases, may lead to a decrease in physiological parameters, although not significantly lower than the controls, suggesting that higher concentrations could begin to induce stress and toxicity effects.

For a general comparison of the PW performance as a foliar fertilizer, Table 3 presents the best results obtained in this study alongside literature reports using different foliar fertilizers on tomato plants with biostimulant effects, such as humic substances, seaweed extracts, and vermicompost. The data highlights that PW_A_ and PW_B_ at 100 mg TOC L^–1^ combined with CF promoted growth responses and chlorophyll index values comparable to, or even superior to, those previously reported in the literature.

**Table 3:** Comparison of the Growth and Development Parameters on Tomato Plants under Different Foliar Fertilizations and Their Respective Effects

Foliar fertilizer	Evaluated parameter	Reported effect	Reference
PW_A_ (T-100F)	Shoot and root length, chlorophyll index and leaf number	11, 23, 60 and 39%	This study
PW_B_ (T-100F)	Shoot and root length, chlorophyll index and leaf number	29, 25, 11 and 60%	This study
Seaweed Extract (*Ascophyllum nodosum* *)* (AN)	root length, chlorophyll index and leaf number	59, 20 and 50%	^79^
Humic-like Substances (HLS_A: T-100F)	shoot length, dry biomass, and chlorophyll index	18, 20 and 14%	^31^
Seaweed Extract *Ascophyllum nodosum* (SES30)	SPAD chlorophyll index	25%	^80^
Biostimulants with mineral fertilizers (Vermicompost)	Shoot length, SPAD chlorophyll index leaf number	5, 54 and 8%	^81^

The results of this study indicate that both PW_A_ and PW_B_, at the tested concentrations, particularly at the lower levels (50 and 100 mg TOC L^–1^), significantly enhanced tomato plant growth. The stimulation of chlorophyll production, plant growth, and potential limitations of PW application are closely linked to its chemical composition, which is determined by both the nature of the feedstock and the operational conditions of the HTC process.

Notably, PW_B_ demonstrated outstanding performance, stimulating plant growth and increasing dry biomass, whereas PW_A_ could be associated with enhanced plant energy metabolism, as suggested by the increase in chlorophyll content, regardless of nutritional supplementation, when compared to the distilled water control and the commercial fertilizer control. These beneficial effects are likely attributable to the presence of soluble organic compounds and nutrients in the PW, which, at moderate concentrations, act as biostimulants. However, at elevated concentrations, phytotoxicity may occur due to the accumulation of potentially inhibitory substances, such as phenolic compounds and organic acids.

These findings highlight the potential of PW as a foliar biostimulant, contributing to the valorization of agro-industrial byproducts, reducing reliance on synthetic fertilizers, and supporting environmentally sustainable agricultural practices. Furthermore, the influence of HTC reaction conditions on the chemical composition of PW emphasizes the importance of process control and dosage optimization to ensure agronomic efficiency and environmental safety.

Future studies could further investigate the physicochemical properties of HTC process waters (PWs), together with plant physiological and biochemical responses, to better elucidate the factors and mechanisms underlying the observed effects on plant growth. In addition, studies incorporating larger sample sizes and independent experimental trials, including field assays, could improve statistical power and provide a more robust assessment of treatment-related responses under different experimental conditions. Such approaches would contribute to a more comprehensive understanding of plant–PW interactions and support the evaluation of their safe and effective use in agricultural systems.

## Supplementary Material



## Data Availability

The data is available at the following address: https://hdl.handle.net/11449/296297.

## Notes

Evaluation of the TOC concentration of PW solutions for molecular fluorescence analysis; physicochemical properties of the Ultisol; chemical characteristics of PW_A_ and PW_B_: analysis of adsorbed water, organic and inorganic contents through TGA analysis; tomato plants after 35 days of growth under experimental conditions; tomato growth and chlorophyll data (mean ± SD) (PDF)

## Abbreviations and Nomenclature

EEM: emission-excitation matrix
FTIR: Fourier transform infrared spectroscopy
HC: hydrochar
HTC: hydrothermal carbonization
FCI: Falker chlorophyll index
PW: process water
TGA: thermogravimetric analysis
TOC: total organic carbon
