# Supplementary material for: Effect of Foliar Application of Process Water from Hydrothermal Carbonization of Sugar-Energy Byproducts on Tomato Plant Growth and Chlorophyll Content
Source: ACS Omega. 2026 Oct 29;11(44):68484–95. doi: 10.1021/acsomega.6c06470 (PMC13756716; doi:10.1021/acsomega.6c06470)
Supplement: Supplementary file 1 [file ao6c06470_si_001.pdf]

SUPPLEMENTARY DATA

Effect of foliar application of process water from hydrothermal carbonization of sugar-energy by-products on tomato plant growth and chlorophyll content

Suelen A. Mondek[a], Gabriela A. Nogueira[a], Isabela C. Constantino[a], Odair P. Ferreira[b], Márcia C. Bisinoti[a], Altair B. Moreira[a*]

[a] São Paulo State University (UNESP), Institute of Biosciences, Humanities and Exact Sciences, Zip code 15054-000 São José do Rio Preto, São Paulo State, Brazil.

[b] State University of Londrina (UEL), Department of Chemistry, Laboratory of Advanced Functional Materials (LaMFA), Celso Garcia Cid Highway, PR 445, Km 380, Zip code 86055-900 Londrina, Paraná State, Brazil.

**Corresponding author*:

E-mail: altair.moreira@unesp.br

Tel: +55 17 3221-2509

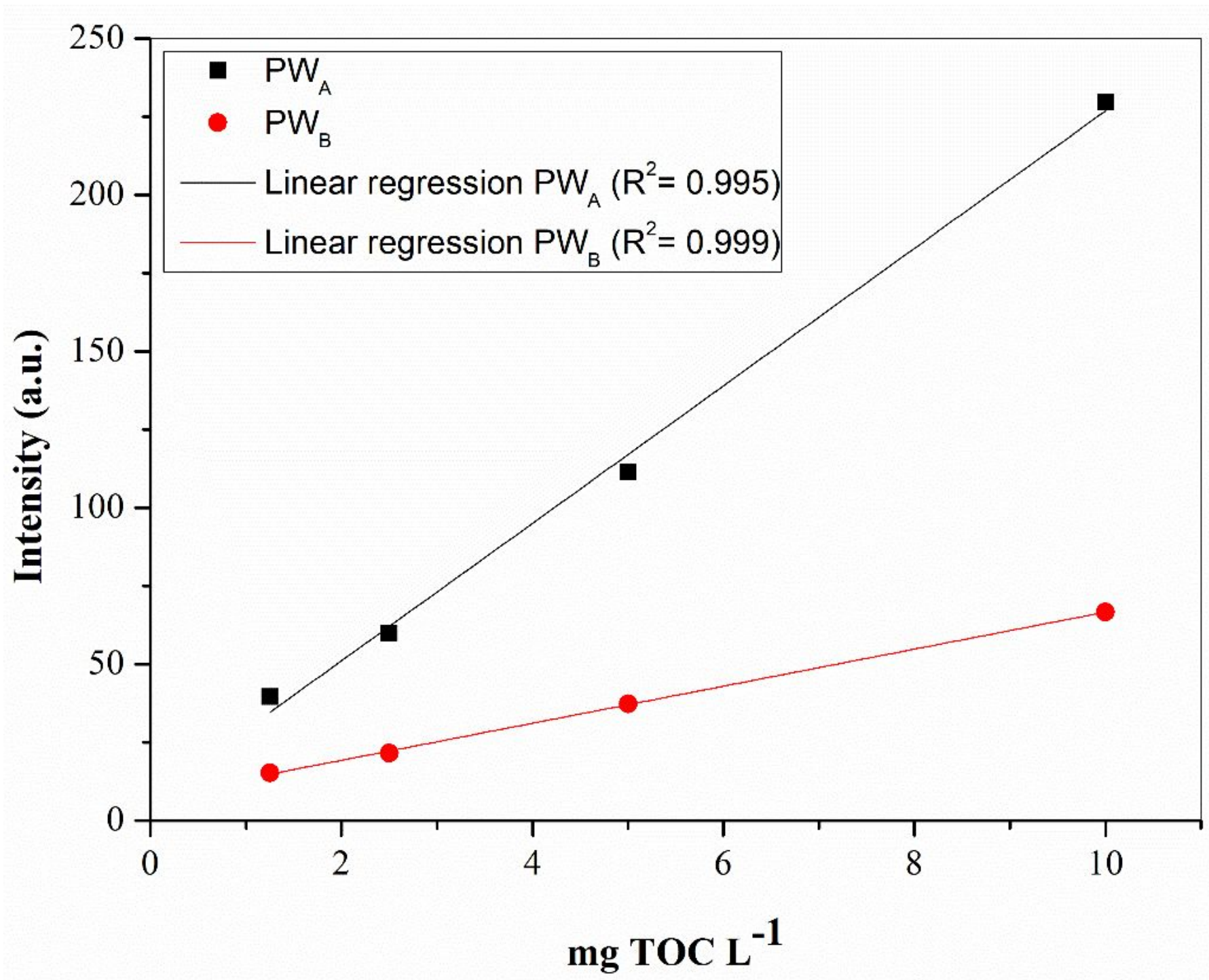

Figure S1. Analytical curve of TOC concentrations of PW solutions for molecular fluorescence analysis in matrix excitation-emission (EEM) mode.

Table S1. Physicochemical properties of the Ultisol used in the tomato plant growth experiment.

**Parameters**	Growth experiment with application	
	PW_A	PW_B**
**OM – organic matter (g dm^{-3})**	16.0	18.7
**pH ($CaCl_2$)***	5.9	4.8
**OC – Organic Carbon (g dm^{-3})**	9.4	10.9
**B (mg dm^{-3})**	0.2	0.2
**Cu (mg dm^{-3})**	0.7	1.7
**Fe (mg dm^{-3})**	14.0	60.0
**Mn (mg dm^{-3})**	10.1	19.5
**Zn (mg dm^{-3})**	3.9	6.4
**CEC - Cation Exchange Capacity (mmol$_c$.dm^{-3})**	55.8	47.7
**Sum of Exchangeable Bases (mmol$_c$.dm^{-3})**	44.5	21.7
**Sand (total) (g kg^{-1})**	904.0	880.0
**Silt (g kg^{-1})**	16.0	80.0
**Clay (g kg^{-1})**	80.0	40.0

* For growth experiments the pH was corrected with liming to pH 6.8. ** PW_B Physicochemical properties[1]

Table S 2. Chemical characteristics of PW_A and PW_B: Analysis of adsorbed water, organic and inorganic contents through TGA Analysis.

Samples	Adsorbed Water (%)[a]	Organic content (%)[b]	Inorganic content (%)[c]
PW_A	7.3	39.2	50.0
PW_B	16.3	72.6	11.1

[a] Percentage mass loss up to 150 °C;
[b] Percentage of mass loss between 150 and 600 °C;
[c] Percentage residue of the TGA curve at 800 °C.

Table S3. Mean values ± standard error of mean (SD) obtained for Chlorophyll Index (FCI) in tomato leaves under treatments applying PW_A and PW_B.

**Treatments**	**Chlorophyll (FCI)**	
	**PW_A**	**PW_B**
**WC**	19.20±0.46	28.30±0.22
**FC**	20.50±1.38	28.96±0.59
**T-50**	30.36±1.79	28.64±0.61
**T-50F**	29.54±0.45	29.86±0.34
**T-100**	30.18±1.35	30.36±0.36
**T-100F**	30.76±0.96	31.48±0.36
**T-300**	27.50±0.57	27.80±0.23
**T-300F**	28.34±0.25	27.68±0.52

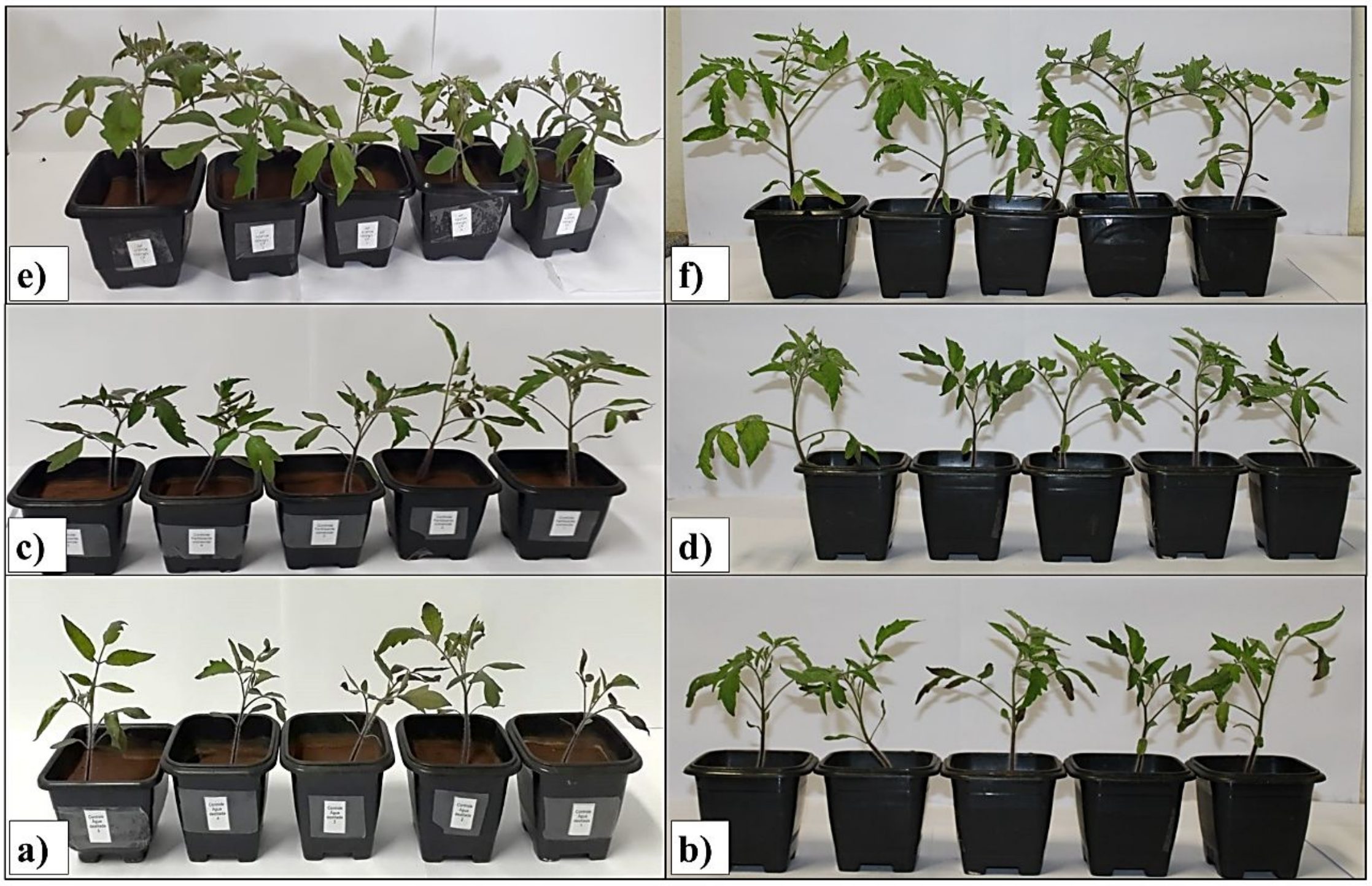

Figure S2. Tomato plants after 35 days of growth under experimental conditions. The 100 mg TOC L^{-1} treatment (T-100F) for both PW_A and PW_B showed the best performance compared to their respective controls: distilled water control (WC) and commercial fertilizer control (FC). PW_A: a) WC; c) FC; e) T-100F. PW_B: b) WC; d) FC; f) T-100F.

Reference

(1) Mondek, S. A.; Constantino, I. C.; Ferreira, O. P.; Bisinoti, M. C.; Miguel, E. C.; Miguel, T. B. A. R.; Metzker, G.; Boscolo, M.; Moreira, A. B. Humic-like Substances Extracted from Hydrochar as an Additive for Foliar Fertilization in Tomato Plants. *ACS Omega* **2025**. https://doi.org/10.1021/acsomega.5c05053.
